# Refractory Lactic Acidosis and Hypoglycemia in a Patient With Metastatic Esophageal Cancer Due to the Warburg Effect

**DOI:** 10.7759/cureus.40563

**Published:** 2023-06-17

**Authors:** Ujjwal Karki, Bijaya Thapa, Shailesh Niroula, Shyam Poudel, Michael Stender, Dilip Khanal

**Affiliations:** 1 Internal Medicine, Corewell Health William Beaumont University Hospital, Royal Oak, USA; 2 Hematology and Oncology, Corewell Health William Beaumont University Hospital, Royal Oak, USA

**Keywords:** case report, esophageal cancer, hypoglycemia, warburg effect, lactic acidosis

## Abstract

The Warburg effect describes a phenomenon in which tumor cells switch their metabolic machinery towards a glycolytic state even in the presence of normal oxygen concentration, resulting in excess lactate production. Lactic acidosis due to the Warburg effect in malignancy is a rare but potentially life-threatening emergency mainly described in hematological malignancies but can occur in non-hematological solid malignancies. To our knowledge, we present the first reported case of lactic acidosis due to the Warburg effect in metastatic esophageal cancer. A 44-year-old male was found to have an esophageal mass and likely hepatic metastases during his hospitalization for altered mental status due to severe hypercalcemia. He was re-admitted two days after discharge for persistent vomiting and an inability to tolerate an oral diet. The lab revealed elevated lactate levels (5.2 mmol/L), metabolic acidosis (pH 7.23), and hypoglycemia (48 mg/dL), all of which were persistent throughout hospitalization despite treatment with intravenous (IV) infusions of dextrose in sodium bicarbonate, IV boluses of dextrose, and IV thiamine. An esophagogastroduodenoscopy with a biopsy of the esophageal mass revealed squamous cell carcinoma of the esophagus. Given the presence of stage IV disease and poor functional status, the patient opted for in-patient hospice, where he passed away. Since prompt diagnosis and initiation of chemotherapy, if possible, are the only effective interventions for this potentially fatal complication, it is important to increase awareness of this underrecognized metabolic and oncologic emergency among physicians.

## Introduction

Lactic acidosis is defined as an elevated serum lactate level greater than 5 mEq/L and a pH less than 7.35 [1]. There are three types of lactic acidosis: type A, type B, and type D, based on the underlying pathophysiology [2]. Type A occurs in the setting of tissue hypoperfusion. Type D is very unusual and occurs in patients with short-bowel syndrome and ingestion of propylene glycol. Type B lactic acidosis occurs in the absence of hypoxemia and tissue hypoperfusion. It can be seen with underlying liver disease, diabetes mellitus, thiamine deficiency, mitochondrial toxins (alcohol, salicylates, reverse transcriptase inhibitors), and malignancies.

Otto Heinrich Warburg first proposed the association between lactic acidosis and malignancy in 1931 [3]. The Warburg effect describes a phenomenon in which tumor cells switch their metabolic machinery towards a glycolytic state even in the presence of normal oxygen concentration, leading to excess lactate production. Type B lactic acidosis in malignancy due to the Warburg effect is rare but associated with a strikingly high mortality rate of over 90% [4]. This phenomenon is mainly described in hematological malignancies, with only a few cases reported involving solid tumors. To our knowledge, we report the first case of type B lactic acidosis in metastatic esophageal cancer.

## Case presentation

A 44-year-old African American male with a recent finding of metastatic malignancy with likely esophageal primary presented with a two-day history of persistent nausea and vomiting and an inability to tolerate an oral diet. He was discharged to a rehabilitation facility three days before the current presentation after a 10-day hospitalization for altered mental status associated with severe hypercalcemia (albumin-corrected serum calcium 17.5 mg/dl; ionized calcium 10.04). At that time, he was found to have an esophageal mass and likely hepatic metastases. Further diagnostic testing was deferred because the patient was cachectic with a body mass index (BMI) of 13.56 kg/m2 and was too frail to undergo any procedure. The severe hypercalcemia, likely from malignancy, resolved after treatment with IV fluids, calcitonin, and pamidronate (albumin-corrected calcium 8.8 mg/dl and ionized calcium 5.22 mg/dl at discharge). His mental status had returned to baseline (normal), and he was tolerating an oral diet before being discharged at that time.

**Figure 1:**
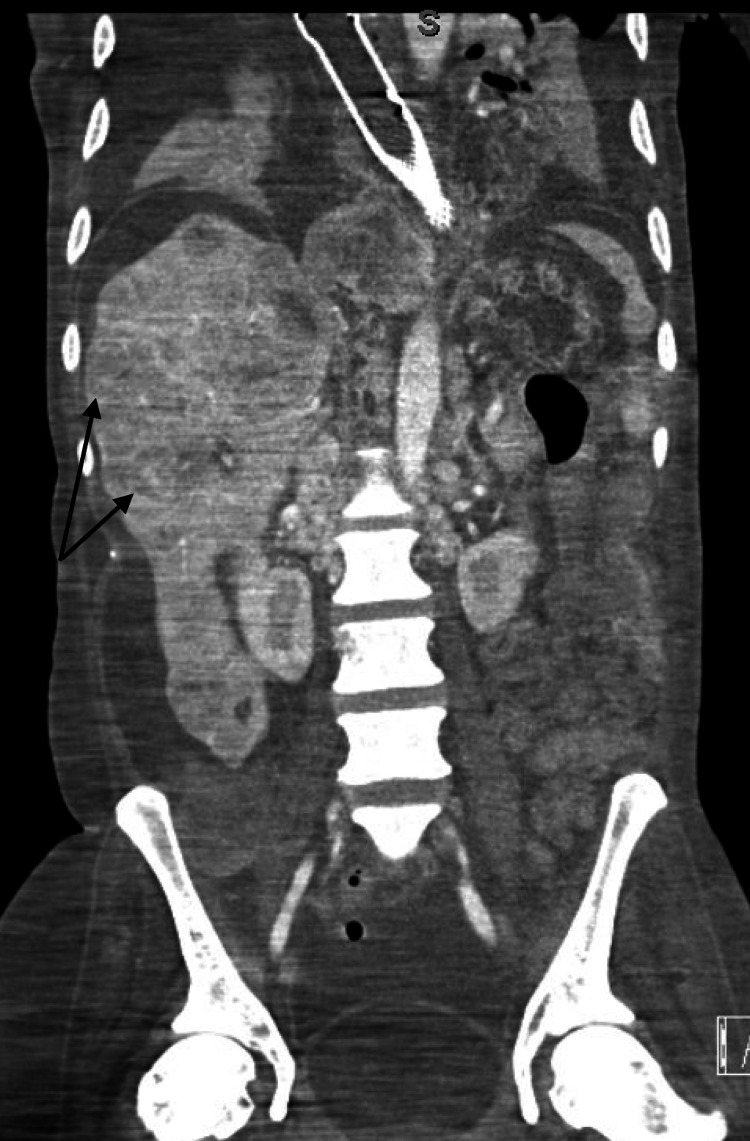
Contrast CT chest/abdomen with arrows showing multiple hepatic metastases

The day after discharge, he started vomiting after every feed and was unable to tolerate both solids and liquids, which led him back to the hospital. Vitals on arrival were notable for blood pressure of 107/75 mm Hg, heart rate of 115 beats per minute, respiratory rate of 16 beats per minute, and oxygen saturation of 100% in room air. On examination, he appeared cachectic and dehydrated. The chest was bilaterally clear to auscultation, and there was mild diffuse abdominal tenderness without guarding, rigidity, or rebound tenderness. Laboratory findings were significant for leukocytosis (12.3 × 109/L), anemia (8.2 g/dl, which was the patient’s baseline), low albumin-corrected calcium (8 mg/dl), normal ionized calcium (4.60 mg/dl), elevated alkaline phosphatase (249 U/L) with otherwise normal liver enzymes and an International Normalized Ratio (INR) of 1.4, hypoalbuminemia (2 g/dl), significantly elevated lactate levels (5.2 mmol/L), and blood glucose of 48 mg/dL. He had high anion gap metabolic acidosis (pH of 7.23, bicarbonate 15 mEq/L, anion gap 18). Blood, urine, and sputum cultures were negative. Ultrasound of the abdomen was suspicious for acute cholecystitis; however, the HIDA scan revealed a patent cystic duct. Computed tomography (CT) of the chest and abdomen showed a previously known necrotic mass measuring up to 6.1 cm in the region of the right crus of the diaphragm, causing leftward deviation of the esophagus with bulky hilar and mediastinal lymphadenopathy and innumerable large hepatic metastases measuring up to 7.3 cm (Figure 1).

Treatment was initiated with dextrose in sodium bicarbonate (NaHCO3, 100ml/hr, intravenously), but lactic acid remained persistently elevated throughout hospitalization with associated persistent hypoglycemia (Table 1) despite being hemodynamically stable (mean arterial pressure above 65mm Hg, systolic blood pressure above 90mm Hg) with no clinical signs of inadequate tissue perfusion. On day two, he was also started on 100 mg per day of intravenous thiamine without any improvement in lactate level.

**Table 1:** Summary of serum lactic acid and glucose during the hospital stay

Hospital day	1	2	3	4	5	6	7	8	9
Lactic acid (mmol/L)	5.2	6.2	4.9	5.1	7.0	7.3	7.7	7.8	5.0
Blood glucose (mg/dl)	48	78	71	111	46	62	61	<10	11

Per the patient's wishes to improve nutrition and pursue treatment if offered to him in the future, an esophagogastroduodenoscopy (EGD) with a biopsy of the esophageal mass was performed on day six. A completely obstructing mass was seen in the middle and lower thirds of the esophagus, where a stent was placed. A biopsy showed squamous cell carcinoma of the esophagus. The patient remained symptomatic with poor tolerance of oral intake. Given the presence of stage IV disease and poor functional status, the patient was a poor candidate for systemic therapy. After extensive discussion, he chose to withdraw any life-prolonging measures and pursue a comfort-based approach. He was transferred to the inpatient hospice unit on day nine, where he passed away the next day (day 10).

## Discussion

Cancer patients are prone to both type A lactic acidosis due to tissue hypoperfusion and type B lactic acidosis due to the Warburg effect. In our patient, there were no signs of infection or hypoperfusion; however, lactic acidosis was persistent and trending upward with associated hypoglycemia. We attributed this to ‘aerobic glycolysis’, a term that succinctly describes the Warburg effect. Type B lactic acidosis is mostly seen in hematological malignancies since it is associated with high cell turnover. However, it has also been described rarely (<50 reported cases) in patients with solid nonhematological tumors, including small cell lung cancer (most common), breast adenocarcinoma, undifferentiated carcinoma, gastric adenocarcinoma, colon cancer, prostate cancer, and gynecological cancers [5-8].

In normal cells, lactic acid is formed under anaerobic conditions from pyruvate, the product of glycolysis, and is metabolized primarily in the liver by gluconeogenesis. Increased lactate production can occur due to the rapid rate of glycolysis by tumor cells secondary to an aberrant insulin-like growth factor (IGF) signaling system that induces overexpression of the enzyme type II hexokinase, which is responsible for catalyzing the first step in glycolysis [4]. Glucose is overutilized by tumor cells and leads to overwhelming glucose consumption, which can lead to hypoglycemia in addition to lactic acidosis, as seen in our patient [9]. Other proposed mechanisms for type B lactic acidosis in malignancy include inadequate lactic acid clearance by the liver due to impaired gluconeogenesis, thiamine deficiency, and local hypoxia due to tumor outgrowing vascular supply [7,10,11].

Lactic acidosis associated with malignancies carries a very poor prognosis with a mortality rate of over 90% and can be considered an oncological emergency [4,5]. However, given the lack of formal prospective trials due to the rarity of the condition, the treatment of type B lactic acidosis associated with malignancy has not been fully established and is mainly based on case reports. Prompt initiation of chemotherapy appears to be the only intervention with a survival benefit as it addresses the underlying etiology by reducing the tumor burden [5,7]. However, like in our case, most patients have advanced cancer and poor functional status, such that they are unable to tolerate antineoplastic interventions. In a review of 13 cases of solid tumor-associated type B lactic acidosis identified from 1998 to 2017, only 23% were able to receive chemotherapy, and they had relatively better survival (months) compared to others (days to weeks) [7].

Supportive management, including intravenous bicarbonate, thiamine supplementation, and renal replacement therapy, is often used as a temporary bridge to reverse the effects of acidemia, but its effect on mortality or lactate concentrations has not been directly studied. In a review by Nair et al., among 18 cases treated with intravenous bicarbonate without hemodialysis, 14 had either no response or worsening of lactic acidosis, and three reported an improvement in acidosis that was transient [5]. The rationale for thiamine use is that it would drive pyruvate towards acetyl coenzyme A synthesis instead of being shunted towards lactic acid; however, some reports suggest thiamine could favor tumor growth [12]. Despite receiving bicarbonate infusions and thiamine supplementation, our patient had persistent lactic acidosis.

## Conclusions

It is essential to report this potentially fatal complication of malignancy to help increase awareness of this underrecognized metabolic, oncologic emergency among physicians. Proposed mechanisms for type B lactic acidosis in malignancy include inadequate liver clearance of lactic acid, aberrant IGF pathways, thiamine deficiency, and local hypoxia. There is no standard of care therapy available; current options include chemotherapy, bicarbonate supplementation, renal replacement therapy, and thiamine supplementation. Early recognition of type B lactic acidosis and prompt initiation of chemotherapy, if feasible, may improve survival.
